# Influence of biological sex, age and smoking on Graves’ orbitopathy – a ten-year tertiary referral center analysis

**DOI:** 10.3389/fendo.2023.1160172

**Published:** 2023-04-04

**Authors:** Michael Oeverhaus, Luisa Winkler, Kerstin Stähr, Anke Daser, Nikolaos Bechrakis, Mareile Stöhr, Ying Chen, Anja Eckstein

**Affiliations:** ^1^ Department of Ophthalmology, University Hospital Essen, Essen, Germany; ^2^ Department of Trauma, Hand and Reconstructive Surgery, University Hospital Essen, Essen, Germany; ^3^ Department of Otorhinolaryngology, Head and Neck Surgery, University Hospital Essen, Essen, Germany

**Keywords:** Graves’ disease, Graves’ orbitopathy, thyroid eye disease (TED), TED, GO, RAI (radioiodine) ablation, sex, age

## Abstract

**Purpose:**

Severity of Graves’ orbitopathy (GO) shows wide individual differences. For optimal treatment, it is important to be able to predict the natural course of the disease as accurate as possible to counteract with anti-inflammatory and surgical treatment. Therefore, we aimed to further elucidate the impact of sex, age and smoking on GO.

**Methods:**

We collected the clinical and demographic data of all patients of our tertiary referral center from January 2008 till December 2018 and analyzed it with descriptive statistics. Only patients with a complete data set were included in the further analysis. Odds ratio’s for moderate-to-severe and sight-threatening GO in relation to age, sex and smoking were calculated by means of multivariate logistic regression models.

**Results:**

We evaluated the data of 4260 patient with GO and complete data sets. Most of these were women (83%). There were no significant differences between male and female patients regarding smoking habits and thyroid treatment. Men were significantly older at initial manifestation of TED (51.8 vs. 49.9y, p<0.01) and showed significant more often severe stages (61% vs. 53%, p<0.0001). Therefore, they needed significantly more intense treatment with steroids, irradiation, orbital decompression and muscle surgery. In multivariate logistic regression analyses age (OR 0.97, 95% CI:0.97-0.98, p<0.0001), male sex (OR 1.64, 95% CI:1.38-1.9, p<0.0001), smoking (OR 1.19, 95% CI:1.04-1.36, p=0.01), Grave’s disease (OR 1.55, 95% CI:1.26-1.90, p<0.0001) and history of radioiodine treatment (RAI) (OR 2.44, 95% CI:2.10-2.86, p<0.0001) showed an significant association with severe stages of GO.

**Discussion:**

Our retrospective analysis showed once more that women are more often afflicted by GO. In contrast, men seem to be more severely afflicted and in need of anti-inflammatory and surgical treatments. This might be due to a different approach to the health system and resilience to GO specific symptoms, as well as previously described worse thyroid control. Estrogen mediated effects might also play a role as in other autoimmune diseases and should be subject of further trials. Besides the biological sex, smoking could again be confirmed as serious risk factor for severe GO. Of note, RAI was associated with more severe stages of GO, which should be subject to further investigation.

## 1 Introduction

Graves’ orbitopathy (GO) is a disorder of autoimmune origin and the most common extrathyroidal manifestation of Graves’ disease (GD) (1–3). The disease is mediated by TSH receptor autoantibodies (TRAb), which stimulate receptors on orbital fibroblasts. In conjunction with the crosstalk with IGF-1 receptors and the activation of several immunomodulatory cells (e.g. T-cells, macrophages) this leads to a cascade of inflammatory conditions (4, 5). The orbital fibroblasts are then stimulated to release inflammatory cytokines, to produce hyaluronic acid and to differentiate into adipocytes and myofibroblasts (6–10). Consequently, patients suffer from signs of soft tissue inflammation (pain, swelling), diplopia (due to fibrosis of extraocular muscles) and proptosis (due to adipogenesis) to a variable extent. Thus, GO has a serious impact on the quality of life of the affected patients (11, 12). Most severe cases develop sight threatening disease mainly due to optic nerve compression (13). The severity and prognosis of GO is reported to be affected by age, biological sex, genetic factors and habitual factors, mainly smoking (14, 15). The prevalence is higher in women, although the female-to-male ratio (F/M) varies depending on the study. However, all studies show that the F/M ratio is lower compared to GD (GD: 3.4-5.6; GO: 2.1-4.2) (16–19). Furthermore, F/M ratio gets lower with higher severity of GO (mild 9.3, moderate 3.2, sight threatening 1.4) (20, 21). This is in concordance with several studies who reported over the years that male GO patients are more severely afflicted (18, 20–25). However, there are also studies showing no significant difference in severity of the disease (26, 27). More recently a GO mouse study also showed no difference in severity between female and male mice, although male mice developed symptoms earlier (28). Clinical studies did not all check for the possible confounding factor of smoking. Smoking is the strongest habitual risk factor and showed in a meta-analysis a 4-fold increased risk of GO occurrence (23, 25, 29, 30). Furthermore, the severity and response to treatment is also known to be worse in smokers (23, 31). Besides smoking and sex, age is also a risk factor: Patients above 60 years seem to be at greater risk of developing severe disease (14). Since previous studies did not have enough statistical power to check for confounding risk factors or simply did not perform such analyses, we aimed to further elucidate the importance of sex, age and smoking on severity of GO. Therefore, we performed subgroup analyses to minimize confounding factors, as well as multivariate logistic regression analyses in our retrospective study in our tertiary GO referral center.

## 2 Patients and methods

### 2.1 Study population

For this retrospective study we retrospectively analyzed data from all patients who visited our EUGOGO (European Group On Graves’ Orbitopathy) tertiary referral center from January 2008 till December 2018 and were referred as GO patients. Only patients with actual diagnosis of GO and complete data sets were included in this study. Baseline characteristics and the course of the disease (treatments, surgeries) were assessed. The retrospective study was performed in accordance with the Declaration of Helsinki and approved by the Ethics Commission of the University of Essen (reference number: 22-10729-BO).

### 2.2 Clinical assessment

Eye examinations were performed at our center using a modified EUGOGO case record form and Color Atlas in a standardized manner (32). All patients were evaluated by a highly trained orthoptist and by one of the specialized ophthalmologists (AE,MO,YC,MS). GO was diagnosed in accordance with the published EUGOGO criteria and mainly based on typical clinical signs (e.g. lid retraction, exophthalmos, restrictive motility disorder, soft tissue involvement) on examination, which was comprised of BCVA, slit-lamp biomicroscopy, applanation tonometry, funduscopy, Hertel exophthalmometry, assessment of subjective diplopia and objective measurement of deviation using the prism-cover-test and measurement of monocular excursions (15). Thyroid disease was categorized into Graves’ disease (active hyperthyroidism or already treated), primary hypothyroidism and euthyroidism (no thyroid disease in follow-up examinations). In case of such an absence of a thyroid disease the clinical signs, MRI or CT images and thyroid specific antibody levels (TRAb, Anti-TPO) were used to diagnose a euthyroid GO. GO activity was evaluated using the CAS (Clinical activity score) classification system established by Mourits et al. (33, 34). GO was classified active with CAS values of ≥3/7 points. Severity assessment was performed according to the EUGOGO guidelines into mild, moderate-to-severe and sight threatening (dysthyroid optic neuropathy [DON] and/or corneal breakdown) (15).

### 2.3 Statistical evaluation

For metric data, median values ( $\tilde{x}$ ) and range or the mean and standard deviation (SD±) were calculated and differences between groups were evaluated with Student’s t-test (two-tailed) if D’Agostino-Pearson omnibus-normality-test showed normal distribution, if not with Mann-Whitney Test. Fisher’s exact test was used to evaluate group distributions of binary variables. Multivariable logistic regression analyses were carried out to evaluate the independent relationship of significant risk factors for severe stages of GO. Here, a first model analyzed the association between age, male sex, smoking, Graves’ disease (vs. hypo-/eutyhroidism) and history of RAI with the occurrence of moderate-to-severe GO. A second model was employed to analyze the association of these covariates with sight-threatening GO. Level of statistical significance was defined two-tailed as 2α<0.05. All calculations were performed with SPSS (IBM SPSS Statistics, Chicago, IL, USA, Version 22.0.0), and Graph Pad Prism (Prism 9 for Windows, Software Inc., San Diego, CA, USA, Version 9.0.0). P-values are given descriptively without α-adjustment for multiple testing.

## 3 Results

### 3.1 Study population

Of all 4641 who were referred to our center as (possible) GO patients, 4381 patients were diagnosed as GO. The other 260 patients who were referred as possible GO showed either unspecific symptoms (slight lid or proptosis asymmetry) or other ocular diseases (e.g., ptosis, strabismus of other origin). After excluding patients with incomplete data sets 4260 patients were included in the analysis. Females were more frequent (n=3502) than men (n=758), resulting in F/M ratio of 4.6. Most patients showed a moderate-to-severe GO (n=2307, 55%), followed by mild cases (n=1777, 41%) and least sight threatening cases (n=176, 4%). The mean age (SD) was 50.3 ± 14 (29-80) years (an overview of baseline characteristics is provided in **Table 1**).

**Table 1:** Characteristics of study population.

	All (n=4260)	Males (n=758)	Females (n=3502)	*p*
Age at onset	50.3 ± 14	51.8 ± 14	49.9 ± 14	0.003^a^
Thyroid disease				
Graves’ disease	89%	87%	89%	0.01^b^
Hypothyroidism	7%	5%	8%	0.004^b^
Eutyhroidism	4%	8%	3%	<0.001^b^
Thyroid treatment				
ATD	40%	44%	39%	0.005^b^
Thyroidectomy	34%	37%	40%	0.32^b^
Primary RAI	24%	21%	25%	0.04^b^
RAI after Tx	5%	3%	5%	0.08
GO status at baseline				
Mild	41%	33%	43%	<0.001^b^
Moderate-to-severe	55%	62%	54%	<0.001^b^
Sight threatening	4%	6%	4%	0.009^b^
Treatment period	13.3 ± 19	12 ± 18	13.5 ± 20	0.17^a^
Smoking status				
Non-smoker	63%	64%	63%	0.14^b^
Smoker	28%	27%	28%	0.53^b^
Past smoker	9%	9%	9%	0.72^b^
Cigarettes per day	13.4 ± 8	14.8 ± 10	13.1 ± 8	0.06^a^

Unless otherwise stated data are means ± SD or proportions (%) or median ( $\tilde{x}$ ) [range]; a: t-test/Mann-Whitney-test, b: Fishers exact test.

### 3.2 Sex specific characteristics

**Figure 1:**
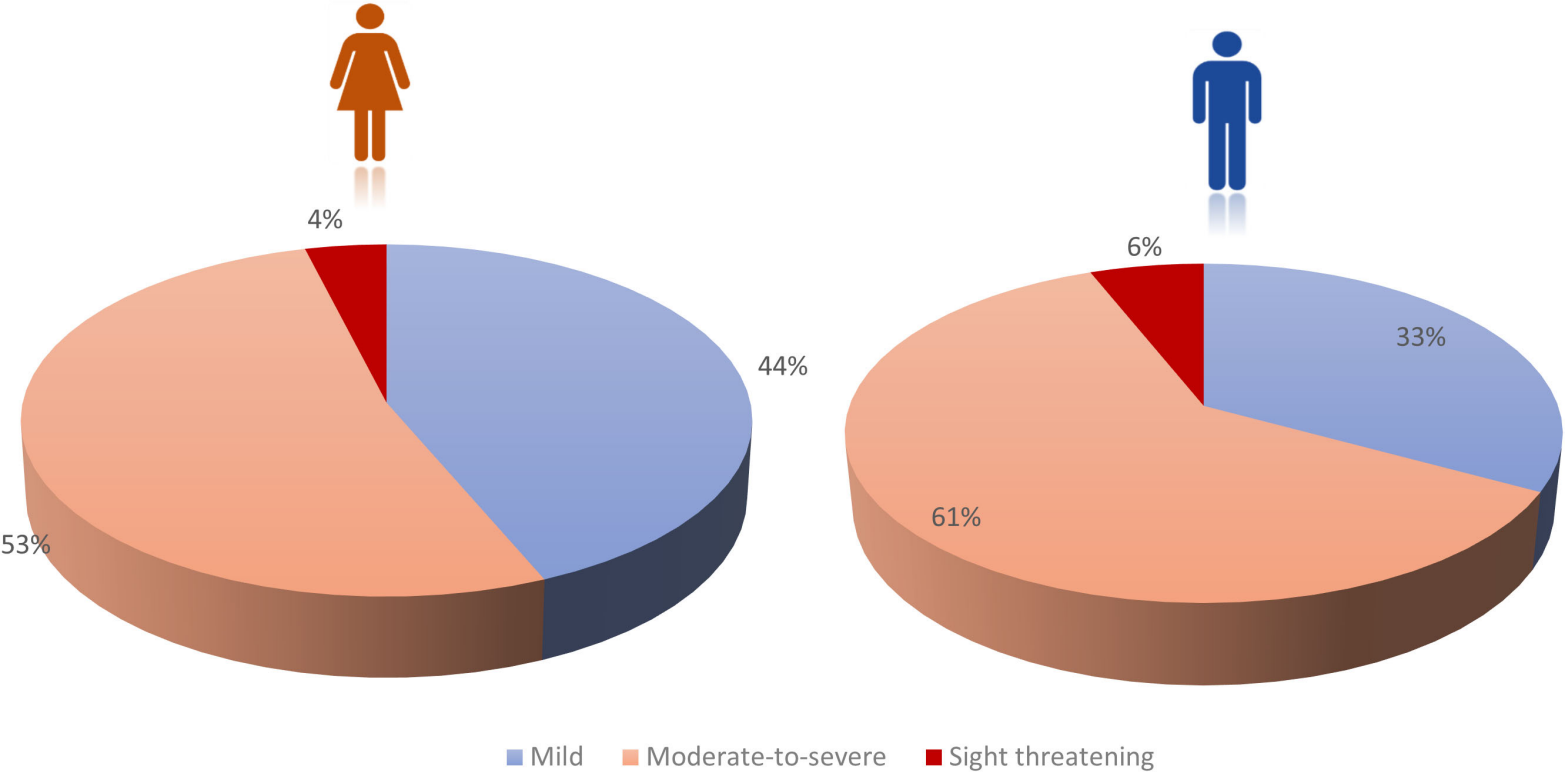
Mild, Moderate-to-severe and Sight-threatening GO in female (n=3502) and male patients (n=758) in a tertiary referral center.

**Figure 2:**
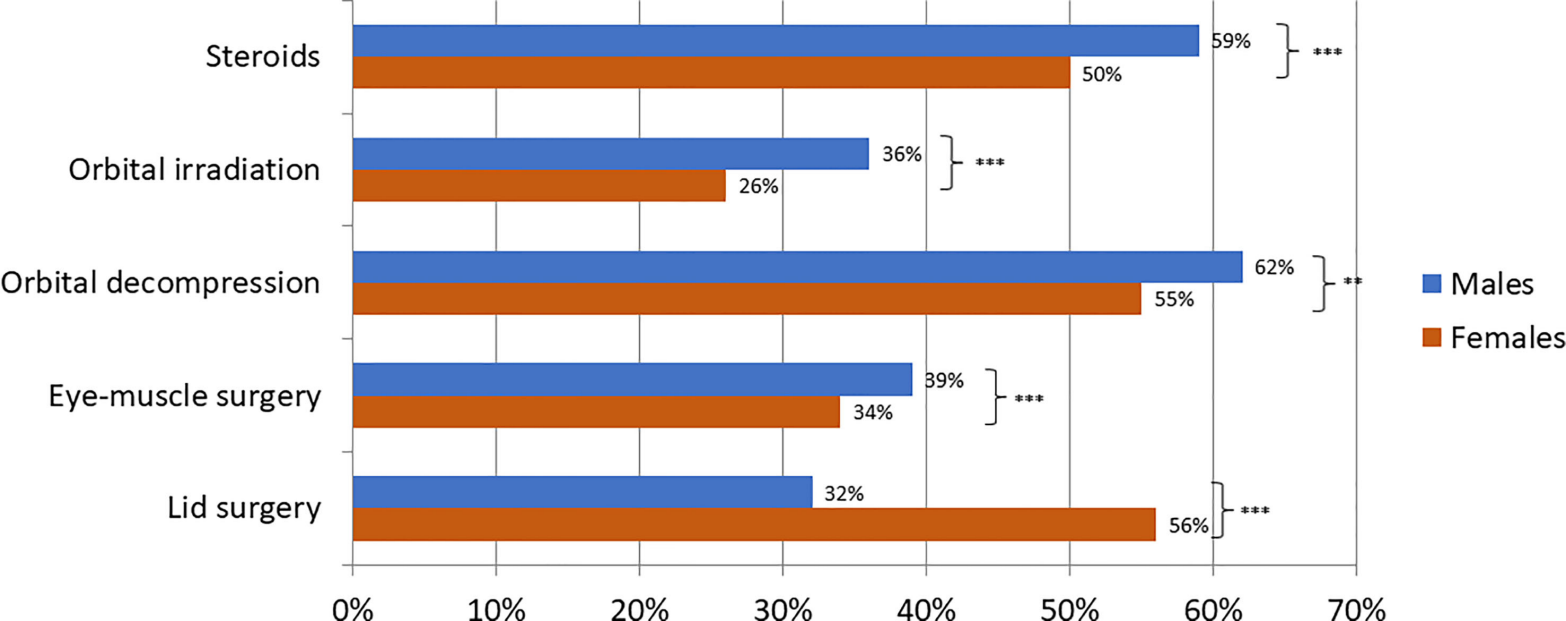
Treatments for GO patients stratified by sex as proportions. (Fisher’s exact test, **<0.01 ***< 0.001).

Our analysis showed already significant differences of the baseline characteristics between males and females: Male patients presented more often with euthyroid and hypothyroid GO, whereas females were significantly more often afflicted by Graves’ disease and were more often treated with antithyroid drugs and radioiodine ablation (see **Table 1**). Mean age at presentation was significantly higher among the male patients, but smoking status showed no significant differences between the two groups. Men showed significantly more often moderate-to-severe and sight threatening GO (see **Figure 1**) and needed consequently more often steroids, orbital irradiation, orbital decompression surgery and rehabilitative muscle surgery (see **Figure 2**). Still, women received more lid surgery (56% vs 32%, p=0.0001).

### 3.3 Smoking subgroup analysis

To further elucidate the influence of smoking we performed a subgroup analysis stratified by smoking status (current smoker vs. non-smoker). Only patients with complete data on the quantity of smoking were included (n=3890). Among non-smokers (n=2696) mild cases were significantly more often, whereas moderate-to-severe cases were significantly more common among smokers (see **Table 2**). Most significant was the difference regarding the occurrence of sight threatening cases, which was much more often in smokers (6% vs. 3%). Furthermore, smokers (n=1194) needed significantly more often orbital decompression as well as steroid treatment, despite being significantly younger. Furthermore, the treatment duration was significantly increased compared to non-smokers. Orbital irradiation, eye muscle and lid surgery showed no significant difference between groups.

**Table 2:** Subgroup analysis of current smokers vs. non-smokers.

	Smoker (n=1194)	Non-smoker (n=2696)	*p*
Age at onset	49.1 ± 11	50.8 ± 15	0.0003^a^
GO status at baseline			
Mild	39%	43%	0.02^b^
Moderate-to-severe	55%	54%	0.02^b^
Sight threatening	6%	3%	0.0001^b^
Treatment period	6.2 [0-107]	4.8 [0-106]	0.04^a^
Treatments			
Steroids	54%	51%	0.04^b^
Orbital irradiation	27%	29%	0.26^b^
Lid-surgery	17%	18%	0.44^b^
Eye muscle surgery	19%	18%	0.62^b^
No. of procedures	1.65	1.64	0.45
Orbital decompression	23%	18%	0.0013^b^

Unless otherwise stated data are means ± SD or proportions (%) or median ( $\tilde{x}$ ) [range]; a: t-test/Mann-Whitney-test, b: Fishers exact test.

To rule out the influence of smoking on differences in GO severity between males and females we further analyzed the smoking subgroups stratified by sex. Among non-smokers male patients (n=484) showed significantly more often moderate-to-severe (59% vs 53%, p=0.01) and less of often mild GO (37% vs 44%, p=0.002 see **Table 3**). Sight threatening cases were more common among males without reaching statistical significance (4% vs 3%, p=0.15). Consequently, male patients needed significantly more often steroids, orbital irradiation, eye muscle and orbital decompression surgery. Furthermore, they needed a significantly higher amount of eye muscle procedures and were significantly older compared to the female patients (53 vs 50 years, p=0.0006). Still, female patients needed significantly more often lid surgery (19% vs. 13%, p=0.001).

**Table 3:** Subgroup analysis of non-smoking male vs. female.

	Male (n=484)	Female (n=2212)	*p*
Age at onset	53.0 ± 15	50.3 ± 15	0.0006
GO status at baseline			
Mild	37%	44%	0.002^b^
Moderate-to-severe	59%	53%	0.014^b^
Sight threatening	4%	3%	0.15^b^
Treatments			
Steroids	55%	50%	0.02^b^
Orbital irradiation	37%	27%	0.0001^b^
Lid-surgery	13%	19%	0.001^b^
Eye muscle surgery	25%	17%	0.0001^b^
No. of procedures	1.75	1.60	0.03^a^
Orbital decompression	23%	18%	0.003^b^

Unless otherwise stated data are means ± SD or proportions (%) or median ( $\tilde{x}$ ) [range]; a: t-test/Mann-Whitney-test, b: Fishers exact test.

In the smoking subgroup there was no significant age difference, but other significant differences in severity between males (n=206) and females (n=989) as displayed in **Supplemental Table 1**. Males suffered significantly more often by moderate-to-severe GO (65% vs 52%, p<0.001), and less often by mild GO compared to females (26% vs 42%, p<0.001). Sight threatening GO showed a markedly higher incidence among males (9% vs 5%, p=0.15), without reaching statistical significance. Males needed consequently significantly more often steroids and orbital irradiation, but surgeries were not significantly different compared to females.

### 3.4 Age and severity

To evaluate the influence of age on severity of GO we performed a subgroup analysis and divided into patients ≥50 and below 50 years. In the older group (n=2246), patients showed significantly more often moderate-to-severe and sight threatening GO (61% vs 47%; 6% vs 3%, p=0.0001, see **Table 4**) and significantly less often mild forms. Steroids, irradiation, lid and orbital surgery were significantly more often required in the older group, in contrast to strabismus surgery which showed no significant difference.

**Table 4:** Subgroup analysis stratified by age (≥50 vs<50 years).

	≥50 (n=2246)	<50 (n=2014)	*p*
Age at onset	60.6 ± 8	38.8 ± 9	0.0001^a^
GO status at baseline			
Mild	33%	50%	0.0001^b^
Moderate-to-severe	61%	47%	0.0001^b^
Sight threatening	6%	3%	0.0001^b^
Treatments			
Steroids	58%	45%	0.0001^b^
Orbital irradiation	36%	20%	0.0001^b^
Lid-surgery	10%	15%	0.0001^b^
Eye muscle surgery	14%	13%	0.27^b^
No. of procedures	1.65	1.66	0.72^a^
Orbital decompression	10%	19%	0.0001^b^

Unless otherwise stated data are means ± SD or proportions (%) or median ( $\tilde{x}$ ) [range]; a: t-test/Mann-Whitney-test, b: Fishers exact test.

### 3.5 Multiple logistic regression

**Figure 3:**
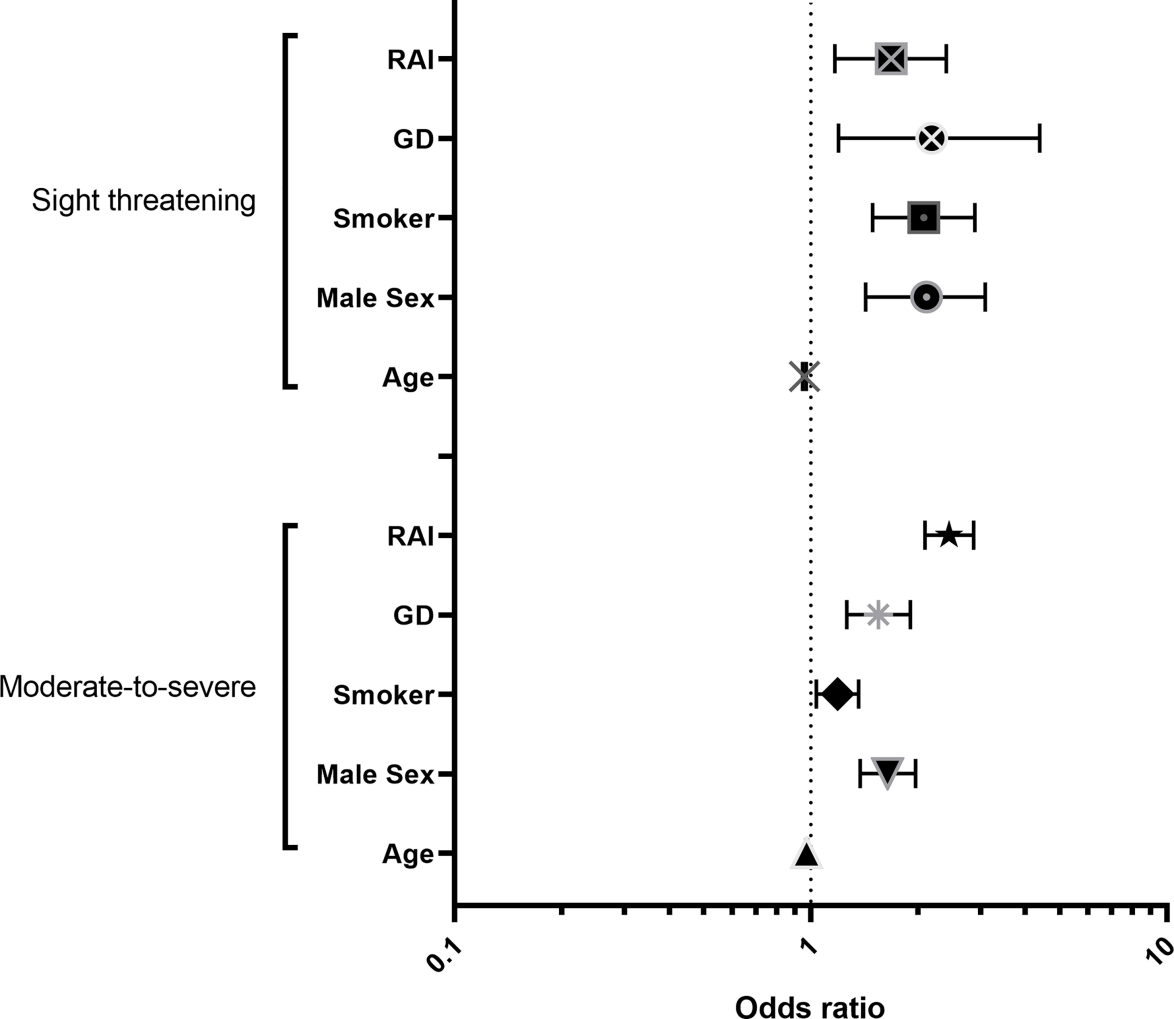
Odds ratio plot for the multiple logistic regression models depicting Odds ratios for the predicting factors and their 95% confidence interval; Model 1 is predicting occurence of moderate-to-severe GO, and Model 2 of sight-threatening GO.

**Figure 4:**
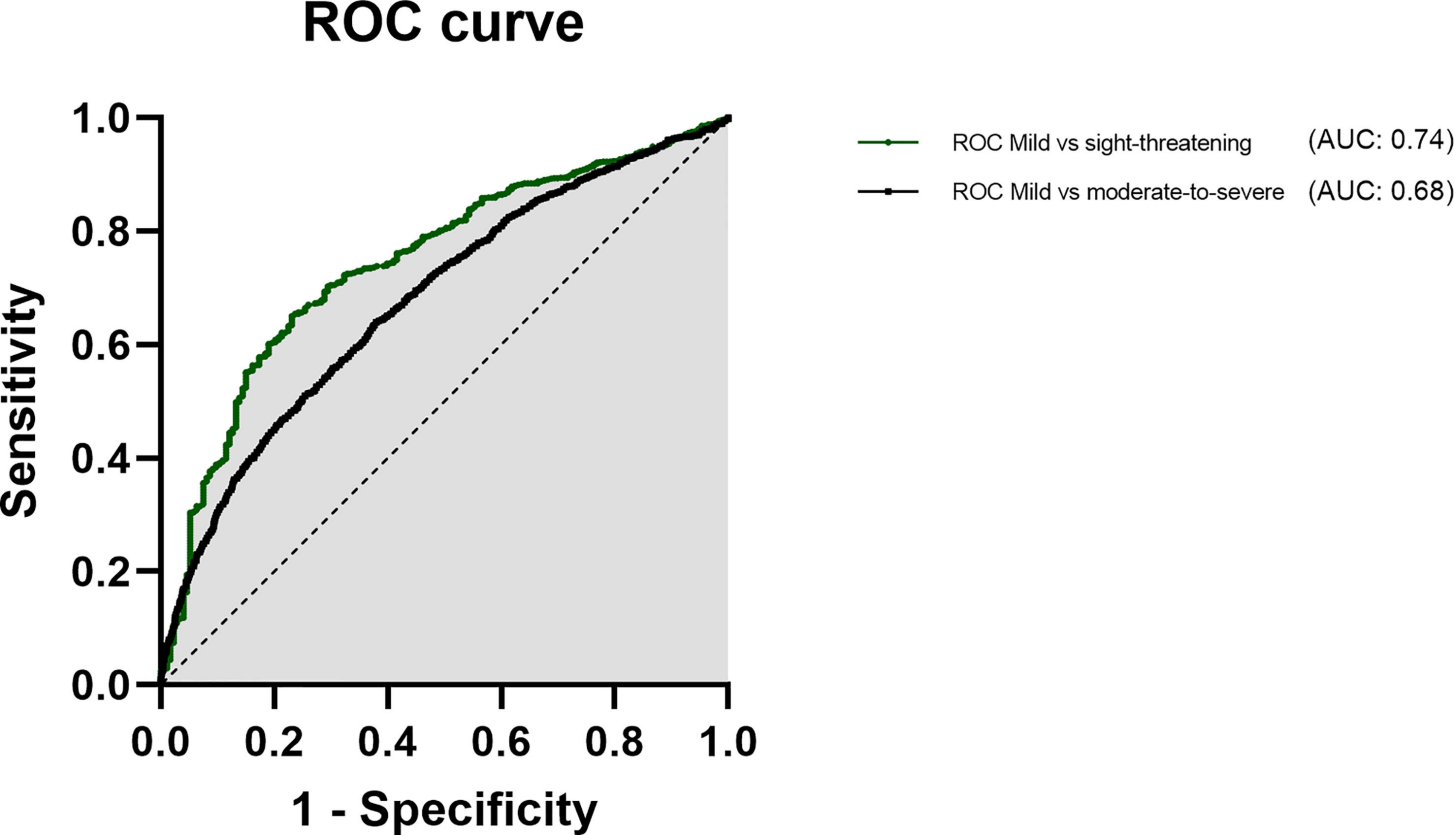
Area under the receiver operating characteristic curve for age, male sex and smoking as predictors for severe (black) and sight threatening GO (green).

To further elucidate the effects of age, sex and smoking we used multivariate logistic regression analyses. Due to their known influence, we added the thyroid disease (GD vs euthyroid and hypothyroid cases) and history of Radioiodine treatment to the multivariate regression. Whereas the first model analyzed the association between these five factors and moderate-to-severe GO, the second model was focused on sight threatening cases (both models used mild cases as default). Model 1 showed that age (OR 0.97, 95% CI: 0.97 to 0.98, p<0.0001), male sex (OR 1.64, 95% CI: 1.38 to 1.97, p<0.0001), smoking (OR 1.19, 95% CI: 1.04 to 1.36, p=0.01), Grave’s disease (OR 1.55, 95% CI: 1.26 to 1.90, p<0.0001) and history of RAI (OR 2.44, 95% CI: 2.10 to 2.86, p<0.0001) are significantly associated with moderate-to-severe GO. The second model showed that age (OR 0.96, 95% CI: 0.94 to 0.97, p<0.0001), male sex (OR 2.11, 95% CI: 1.43 to 3.07, p<0.0001), smoking (OR 2.07, 95% CI: 1.49 to 2.89, p=0.0001), Grave’s disease (OR 2.18, 95% CI: 1.19 to 4.39, p=0.018) and history of RAI (OR 1.68, 95% CI: 1.16 to 2.30, p=0.005) are associated also with sight threatening GO (see **Figure 3**). Multicollinearity analysis was employed to ensure the independence of the three variables, which was the case in both models. Goodness-of fit analysis showed a Nagelkerke’s R² of 0.12 for both and a Log-likelihood ratio (G squared) of 377.4 and 106.7, respectively (both p<0.0001) indicating a good prediction model. The Area under the receiver operating characteristic curve (AUC) was observed as 0.68 (95% CI: 0.66 to 0.69) for Model 1 and 0.74 (95% CI: 0.71 to 0.78, see **Figure 4**).

## 4 Discussion

The results of this retrospective study in our tertiary referral center show a higher risk of developing a more severe GO for men and smokers which is in concordance with many previous studies (18, 20–24, 29–31). These patients should be critically monitored and patients vigorously encouraged to stop smoking.

### 4.1 Influence of biological sex on severity of GO

In our retrospective analysis of a large single-center cohort female GO patients were much more common as in previous studies and similar to recent reports of tertial referral centers (PREGO III: 79.2% females vs. 82.2% here) (35). Our evaluation reaffirms the association of male sex with more severe stages of GO (18, 20–25). This in contrast to Kavoussi et al. (26) who reported in a chart review of 62 patients no significant difference in NOSPECS and CAS between men and women (26). Only difference they found was that men showed more often asymmetric disease and exophthalmos than females. Furthermore, an Australian case-control study by Khong et al. (27) reported no significant association of sex and development of GO in 1004 patients in simple and multiple logistic regression models (27). In contrast to our study they compared GO patients (n=604) to GD patients without GO. They did not report how many patients showed mild and moderate-to-severe stages. Assuming a typical distribution (77% mild Tanda 2013 et al.) - their lack of a significant association might be due to mostly mild cases, which seem not to be associated with the male sex (36).

Interestingly, there were in our cohort no significant differences in the needed treatments between male and female non-smokers despite the more moderate-to-severe cases among men. This might indicate that there is a different approach to the health system or even a different resilience to the same symptoms. Women might suffer more under lid related symptoms which might explain that there were even more Lid surgeries among women in this subgroup. Indeed a study by Ponto et al. (37) reported that men were less willing to accept long distances to the center despite being more affected (37). Furthermore, they reported that significantly more women used psychosocial support, which might indicate that female patients suffer by a greater impact on quality of life. However, quality of life studies did not report QoL values divided by sex, which is why this aspect should be evaluated in future prospective studies. However, there have been reports that men demonstrate decreased cosmetic impetus for medical treatment compared to females (14). In addition to the reported reluctance to accept longer access routes, male GD patients have reportedly a worse compliance and follow-up situation, which is why their response to treatment of hyperthyroidism is reportedly worse (38). Since the effect of uncontrolled thyroid status on GO is well known, this might also explain the sex different severity of GO. Furthermore, Radioiodine treatment is significantly more often used in females and a known risk factor in case of insufficient prophylaxis for progression of GO (15, 39). Still, our multiple logistic regression revealed a significant association of sex despite including thyroid status and RAI, which independently contributed to the development of severe GO. However, our study did not included TRab and thyroid hormone levels, which is why the effect of an uncontrolled thyroid more often present in men cannot be ruled out. Another possible explanation might be the effect of estrogen on inflammatory processes as suggested (40). In other autoimmune diseases this effect has already been shown, e.g. in Systemic sclerosis a female predominant disease also showing more severe cases in men (41). However, this theory has not been tested in GO, yet. In conclusion, men have a greater risk of developing moderate-to-severe and sight-threatening GO and should be carefully monitored and even more encouraged to regularly follow-up and stop smoking. Further experimental and prospective studies are needed to further elucidate the reasons for this predisposition of men for more severe GO.

### 4.2 Influence of smoking

In our analysis we confirmed the significant association of smoking with a more severe course of GO as reported before in clinical and experimental studies (23, 29–31, 42). As in a recent EUGOGO report about a quarter of patients were current smokers (24.2% vs 28% here) (35). Our multivariate logistic regression showed a significant association but lower Odd’s ratios (OR= 1.2 and 2.1, respectively) compared to Lee et al. (43), who reported smoking as a predictive risk factor for a severe course of GO and the development of optic neuropathy (OR = 6.57 and 10.00, respectively) in a cohort of 99 patients (43). In contrast, other factors such as age, gender, free T4 level, thyroid binding-inhibiting immunoglobulin, and a history of diabetes were not predictive of severe GO or optic neuropathy in their study. A systematic review concluded that all evidence supports the theory of a causal link, because of the constant association of smoking with the occurrence of GO across all studies, a dose–response effect, a reduced risk of GO in ex-smokers, and the reported temporal relationship (44). Still, most studies focused on the incidence of GO reporting in comparison to GD patients OR between 1.94 and 10.1, and in comparison to control subjects without thyroid disease OR between 1.22 and 20.2. Only 2 studies investigated the association with severe GO (27, 43). In contrast to Lee et al., Khong et al. (27) reported in their Australian cohort (n=1004) a significant association of smoking with the occurrence of GO but not with development of DON. This is in contrast with our results showing smoking to be significantly associated with the occurrence of sight-threatening GO and showing higher OR in the multiple logistic regression compared to moderate-to-severe GO, indicating a larger effect on development of DON. To conclude, smoking is as demonstrated before significantly associated with the development of GO and patients should be advised to quit smoking. In addition, smoking appears to be a risk factor for sight-threatening GO.

### 4.3 Influence of age

In our study the mean age was comparable to other reports (50.3y vs. 50.5y in PREGOIII) (35). However, the influence of age was less pronounced compared to previous reports. Whereas, the median age of more severe stages was higher and the subgroup analysis showed more severe stages in the older group, the multiple logistic regression analyses showed surprisingly younger patients slightly more at risk. This association was smaller compared to smoking and sex. Similar results were found by Woo et al. (45) among 1,632 dysthyroid patients (45). They reported as result from multiple logistic regression analyses that young age, Graves’ disease, dermopathy, anti-thyroid medication treatment, and radioiodine treatment were independent risk factors for the occurrence of thyroid eye disease. However, the study did not differ between different severity stages as in our report. This might be linked to a lower remission rate of younger patients after antithyroid medication (38). Lee et al. (43) reported similarly to our results that patients with more severe GO were older, but no significant association in multiple logistic regression. Smoking was there the only significant predictive factor (43). In conclusion, age seems to have a smaller association to severe stages of GO than sex, smoking and thyroid status.

### 4.4 Multiple logistic regression and further influential factors

The AUC for both models indicate unsurprisingly, that there are further influential factors. Still, the model performed pretty well (AUC 0.68 and 0.74) considering, that only basic factors were included. The addition of TRAb and thyroid hormone levels could further improve the models. RAI had a significant association with moderate-to-severe GO and sight-threatening GO patients as described before (46). The main reason for this association is certainly the fact that patients with poorly controlled thyroid function are of course send for definitive therapy. And these are usually the patients who are at risk for a more severe course of GO (Wiersinga 2018). However Radioiodine therapy itself can be followed by a deterioration or new onset of GO (Törring 1996, Traisk 2009, Dederichs 2006, Bartalena 1997). It has been published that this can be prevented in most of the cases by steroid prophylaxis (Bartalena 1997). However GO can deteriorate in some cases especially with high TRAb and recurrence of hyperthyroidism (Vanucci 2019) and of course also with insufficient prophylaxis for progression of GO (15, 38). The increase of TRAb levels after radioiodine Therapy (Laurberg 2008) is discussed as a major factor for deterioration of GO. Previous reports showed a high association in simple logistic regression models, but no significant in multiple logistic regression when combined with age, smoking and antithyroid medication (27). This might indicate, that RAI is no in general harmful (with sufficient steroid prophylaxis) but should not be considered in patients presenting further risk factors. In our cohort most patients suffered as in previous reports of GD (89% vs 89.9 PREGO III) (35), whereas hypothyroid and euthyroid cases comprised the remaining 11%. The latter seem not be associated with more severe GO, but might be associated with a delayed diagnosis of GO.

## 5 Limitations

Our results could possibly be confounded by other factors such as genetic, ethnic, thyroid related and possibly further unknown associated factors. Still, genetics seem to have the lesser role and our population was mainly Caucasian (47). Furthermore, one should note that our center has an ophthalmological focus, which might be why the cohort comprised of more moderate-to-severe cases than in PREGO III report of other tertiary referral centers (55% vs 39.9%) (35). This is also why LDL cholesterol levels where no regularly assessed, though there are a known risk factor (48). In addition, GD duration was not part of the model, despite a significant role in a previous study (27).

## 6 Conclusion

We could show in our large cohort comprised of 4260 patients of a single tertiary referral center that there is a strong association of male sex, smoking and RAI with more severe stages of GO. Besides stable euthyroidism, cessation of smoking should be a primary goal especially for male patients to avoid severe GO and consecutive medical and surgical treatments.

## Data availability statement

The raw data supporting the conclusions of this article will be made available by the authors, without undue reservation.

## Ethics statement

The studies involving human participants were reviewed and approved by Ethikkommission Universitätsklinikum Essen. Written informed consent for participation was not required for this study in accordance with the national legislation and the institutional requirements.

## Author contributions

Conceptualization: MO, AE, and YC. Methodology: MO, AE, YC, and MS. Software: MO. Validation: MO, AE, YC, LW, NB, AD, and KS. Formal analysis: MO, AE, and LW. Investigation: MO, KS, AD, MS, AE, and YC. Resources: NB, AE, and MO. Data curation: MO and LW. Writing-original draft preparation: MO, AE, and YC. Writing-review and editing: MO, LW, AD, MS, KS, NB, AE, YC. Visualization: MO and LW. Supervision: NB, AE, MO. Project ad-ministration: LW, MS, and MO. Funding acquisition: MO, AE, and NB. All authors have read and agreed to the published version of the manuscript.
