# Supplementary material for: Influence of biological sex, age and smoking on Graves’ orbitopathy – a ten-year tertiary referral center analysis
Source: Front Endocrinol (Lausanne). 2023 Apr 4;14:1160172. doi: 10.3389/fendo.2023.1160172 (PMC10110835; doi:10.3389/fendo.2023.1160172)
Supplement: Supplementary file 1 [file Table_1.docx]

**Supplemental Table 1:** Subgroup analysis smoking male vs. female

|  | Male (n=206) | Female (n=989) | *p* |  |
| --- | --- | --- | --- | --- |
| Age at onset | 49.2 ±12 | 49.2 ±11 | 0.66 |  |
| GO status at baseline |  |  |  |  |
| Mild | 26% | 42% | 0.0001^b^ |  |
| Moderate-to-severe | 70% | 56% | 0.0001^b^ |  |
| Sight threatening | 4% | 2% | 0.09^b^ |  |
| Treatments |  |  |  |  |
| Steroids | 65% | 51% | 0.0003^b^ |  |
| Orbital irradiation | 35% | 25% | 0.0044^b^ |  |
| Lid-surgery | 17% | 18% | 0.15^b^ |  |
| Eye muscle surgery | 19% | 18% | 0.33^b^ |  |
| No. of procedures | 1.78 | 1.62 | 0.31 |  |
| Orbital decompression | 23% | 18% | 0.12^b^ |  |

Unless otherwise stated data are means ±SD or proportions (%) or median ($\tilde{x})$ [range]; a: t-test/ Mann-Whitney-test, b: Fishers exact test
